# Supplementary material for: Multimode ultrastrong coupling in three-dimensional photonic-crystal cavities
Source: Nat Commun. 2025 Apr 16;16:3603. doi: 10.1038/s41467-025-58835-x (PMC12000563; doi:10.1038/s41467-025-58835-x)
Supplement: Supplementary file 1 — Supplementary Information [file 41467_2025_58835_MOESM1_ESM.pdf]

Supplementary Information: Multimode ultrastrong coupling in three-dimensional photonic-crystal cavities

Fuyang Tay[1,2], Ali Mojibpour[1], Stephen Sanders[1], Shuang Liang[3,4], Hongjing Xu[5], Geoff C. Gardner[6], Andrey Baydin[1,7,8], Michael J. Manfra[3,4,6,9], Alessandro Alabastri[1,7,8], David Hagenmüller[10], Junichiro Kono[1,5,7,8,11*]

[1]Department of Electrical and Computer Engineering, Rice University, Houston, Texas 77005, USA.
[2]Applied Physics Graduate Program, Smalley–Curl Institute, Rice University, Houston, Texas 77005, USA.
[3]Department of Physics and Astronomy, Purdue University, West Lafayette, Indiana 47907, USA.
[4]Birck Nanotechnology Center, Purdue University, West Lafayette, Indiana 47907, USA.
[5]Department of Physics and Astronomy, Rice University, Houston, Texas 77005, USA.
[6]School of Electrical and Computer Engineering, Purdue University, West Lafayette, Indiana 47907, USA.
[7]Smalley–Curl Institute, Rice University, Houston, Texas 77005, USA.
[8]Rice Advanced Materials Institute, Rice University, Houston, Texas 77005, USA.
[9]School of Materials Engineering, Purdue University, West Lafayette, Indiana 47907, USA.
[10]CESQ-ISIS (UMR 7006), Université de Strasbourg and CNRS, Strasbourg, 67000, France.
[11]Department of Materials Science and NanoEngineering, Rice University, Houston, Texas 77005, USA.

*Corresponding author(s). E-mail(s): kono@rice.edu;

Contents

1 Theory **2**
- 1.1 Microscopic model . . . 2
- 1.2 Contributions of the different terms in the full Hamiltonian to the ground-state correlations . . . 6
- 1.3 Vacuum fluctuations and localization of the cavity modes . . . 7

2 Numerical simulations **10**
- 2.1 Details of simulations . . . 10
- 2.2 Optimized thickness of the defect layer in numerical calculations . . . 11
- 2.3 Simulations with different ranges of time delays . . . 12
- 2.4 Simulations of a bare 3D-PCC with varying defect layer thicknesses . . . 12

3 Experiment **13**
- 3.1 Noise floor of the THz setup . . . 13
- 3.2 Bare cyclotron resonance . . . 13
- 3.3 Microscope images . . . 14
- 3.4 Transmittance spectra of the bare 3D-PCC . . . 14
- 3.5 Extraction of peak frequencies . . . 17
- 3.6 Error bars of the extracted peak frequencies . . . 22

S1 Theory

S1.1 Microscopic model

The microscopic Hamiltonian is derived in the Coulomb gauge by extending the models of Refs. [1–3]. We use the vector potential operator

$$\hat{\mathbf{A}}(\boldsymbol{\rho}, z) = \sum_{p,\sigma} \sqrt{\frac{\hbar}{2\varepsilon_0 \omega_p a^3}}\, \mathbf{E}_{p,\sigma}(\boldsymbol{\rho}, z) \left(\hat{a}_{p,\sigma} + \hat{a}^\dagger_{p,\sigma}\right). \tag{S1}$$

At normal incidence (Γ point of the first Brillouin zone), the electric field mode functions $\mathbf{E}_{p,\sigma}(\boldsymbol{\rho}, z)$ can be chosen real without loss of generality. The calculation can be trivially extended to the full Brillouin zone using complex fields with a quasi-momentum $\mathbf{q}$ as computed by FDTD. The linear coupling term between light and matter reads

$$\frac{e}{m_{\text{eff}}} \int d\boldsymbol{\rho} dz\, \hat{\Psi}^\dagger(\boldsymbol{\rho}, z) \hat{\boldsymbol{\pi}} \cdot \hat{\mathbf{A}}(\boldsymbol{\rho}, z) \hat{\Psi}(\boldsymbol{\rho}, z), \tag{S2}$$

with $\hat{\boldsymbol{\pi}}$ the gauge-invariant in-plane momentum including the contribution of the static magnetic field B, and the fermion field $\hat{\Psi}(\boldsymbol{\rho}, z) = L^{-1/2} \sum_{n,k} e^{-iky} \chi_{n,k}(x) \xi(z) \hat{c}_{n,k}$ [1]. We use the Landau gauge, where single electron states are characterized by an integer

n and the y component of the electron momentum k. The wave functions are plane waves in the y direction (L is the length of the 2DEG in the plane), and those of a harmonic oscillator (Hermite polynomial of order n) centered at the guiding center position $k\,l_\mathrm{c}^2$, with $l_\mathrm{c} = \sqrt{\hbar/eB}$ the magnetic length. $\xi(z)$ is a normalized wave function that describes the QW confinement in the z direction. $c^\dagger_{n,k}$ and $c_{n,k}$ are the creation and annihilation operators of an electron in the state (n, k).

When computing the matrix elements entering Eq. (S2), we assume that mode spatial profiles $\mathbf{E}_{p,\sigma}(\boldsymbol{\rho}, z)$ remains constant over the QW thickness, and exploit the discrete translational invariance in the plane by decomposing the mode profiles into Fourier series $\mathbf{E}_{p,\sigma}(\boldsymbol{\rho}, z_\mathrm{2DEG}) = \sum_\mathbf{G} \mathbf{U}_{p,\sigma}(\mathbf{G}) e^{i\mathbf{G}\cdot\boldsymbol{\rho}}$, with $\mathbf{G} = (2\pi m_x/a)\mathbf{e}_x + (2\pi m_y/a)\mathbf{e}_y$ ($m_x, m_y \in \mathbb{N}$) the reciprocal lattice vectors. One is then left with the calculation of overlap integrals of the kind

$$I_{n,k}^{n'\pm1,k'}(\mathbf{G}) = \int \frac{dy}{L}\, e^{i(k-k'+G_y)y} \int dx\, \chi_{n,k}(x) \chi_{n'\pm1,k'}(x) e^{iG_x x}. \tag{S3}$$

While the integral in the y direction simply provides the selection rule $k' = k + G_y$, the calculation of the integral along x is performed using the dipole approximation. The mode functions $\mathbf{E}_{p,\sigma}(\boldsymbol{\rho}, z_\mathrm{2DEG})$ are slowly varying over the typical extent of the harmonic oscillator wave functions $\chi_{n,k}(x)$. For a magnetic field $B \simeq 1\,\mathrm{T}$, the latter is indeed $\sim l_\mathrm{c} \simeq 10-100\,\mathrm{nm}$, which is much smaller than the woodpile lattice parameter $a = 333\,\mu\mathrm{m}$. Since the Fourier coefficients $\mathbf{U}_{p,\sigma}(\mathbf{G})$ exhibit large peaks at $G_j \sim 1/a$ ($m_x \sim m_y \sim 1$) and rapidly decrease as $G_j a \to \infty$, one has $G_j l_\mathrm{c} \sim l_\mathrm{c}/a \ll 1$. Equation (S3) thus provides $I_{n,k}^{n'\pm1,k'}(\mathbf{G}) \approx \delta_{k',k+G_y} \delta_{n,n'\pm1} e^{iG_x k l_\mathrm{c}^2}$.

We introduce the CR excitation creation operator

$$\hat{b}^\dagger(\boldsymbol{\rho}) = \frac{1}{a\sqrt{\mathcal{N}}} \sum_{k,\mathbf{G}} \hat{c}^\dagger_{\nu,k-G_y} \hat{c}_{\nu-1,k} e^{iG_x k l_\mathrm{c}^2} e^{-i\mathbf{G}\cdot\boldsymbol{\rho}}, \tag{S4}$$

which promotes an electron with momentum k in the highest-occupied Landau level (LL) $n = \nu - 1$ to the lowest-unoccupied LL $n = \nu$ and momentum $k - G_y$. $\nu = 2\pi n_\mathrm{e} l_\mathrm{c}^2$ denotes the filling factor of the 2DEG and $\mathcal{N}$ is the LL degeneracy. The in-plane position vector $\boldsymbol{\rho}$ is restricted to a woodpile unit cell. The CR excitation operators $\hat{b}(\boldsymbol{\rho})$ and $\hat{b}^\dagger(\boldsymbol{\rho})$ satisfy bosonic commutation relations when the number of CR excitations remains small compared to $\mathcal{N}$, i.e., $\langle[\hat{b}(\boldsymbol{\rho}), \hat{b}^\dagger(\boldsymbol{\rho}')]\rangle = \delta(\boldsymbol{\rho} - \boldsymbol{\rho}')$, where $\langle\cdots\rangle$ denotes the expectation value in the electronic ground state (in which the lowest ν LLs are fully occupied). Note that the LL degeneracy entering the definition of the CR excitation operators is here written as $\mathcal{N} = a^2/(2\pi l_\mathrm{c}^2)$, using the lattice constant a instead of the length L of the 2DEG. This is indeed the only physical choice as the light–matter coupling strength should not depend explicitly on any length scale other than l_c in the plane. With those definitions, one recovers the light-matter coupling term H_int discussed in the main text. The A^2 term is derived in a similar fashion.

The transmission spectra shown in the main text were computed using an input–output model, which is an extension of the one introduced in Ref. [4] in a simple planar geometry. Transmission of THz radiation through the 3D-PCC is modeled by

introducing two identical photon reservoirs on each side of the cavity in the z direction (top, bottom). Similarly, CR excitations of the 2DEG acquire a finite lifetime by interacting with a phenomenological bosonic reservoir. The total Hamiltonian includes the contribution (neglecting counter-rotating terms)

$$\hat{H}_{\mathrm{R}} = \sum_{p,\sigma,\lambda} \int dq\, w_{p,\sigma}(q) \hat{\alpha}^{\dagger}_{p,\sigma,\lambda}(q) \hat{\alpha}_{p,\sigma,\lambda}(q) + i\kappa_{p,\sigma}(q) \left[\hat{\alpha}_{p,\sigma,\lambda}(q) \hat{a}^{\dagger}_{p,\sigma} - \hat{a}_{p,\sigma} \hat{\alpha}^{\dagger}_{p,\sigma,\lambda}(q) \right] \\ + \int dq \int d\boldsymbol{\rho}\, \widetilde{w}(q,\boldsymbol{\rho}) \hat{\beta}^{\dagger}(\boldsymbol{\rho},q) \hat{\beta}(\boldsymbol{\rho},q) + i\widetilde{\kappa}(\boldsymbol{\rho},q) \left[\hat{\beta}(\boldsymbol{\rho},q) \hat{b}^{\dagger}(\boldsymbol{\rho}) - \hat{b}(\boldsymbol{\rho}) \hat{\beta}^{\dagger}(\boldsymbol{\rho},q) \right]. \quad \text{(S5)}$$

The first contributions in the first and second lines of Eq. (S5) describe the energy of the photonic modes in each reservoir $\lambda = (\mathrm{top}, \mathrm{bot})$ and that of the matter excitation reservoir, respectively. q denotes a phenomenological (continuous) parameter ensuring that the reservoirs have continuous spectra. The second terms in both lines describe the coupling between the mode of the reservoirs and the modes of the system, which provides the latter with a finite lifetime. The input–output method consists in solving the equations of motion of the system by introducing "input" and "output" operators: $\hat{\alpha}^{\mathrm{in}}_{p,\sigma,\lambda}(q) = \lim_{t\to-\infty} \hat{\alpha}_{p,\sigma,\lambda}(q,t) e^{iw_{p,\sigma}(q)t}$, $\hat{\alpha}^{\mathrm{out}}_{p,\sigma,\lambda}(q) = \lim_{t\to+\infty} \hat{\alpha}_{p,\sigma,\lambda}(q,t) e^{iw_{p,\sigma}(q)t}$, and similar expressions for the matter excitation reservoir operators. One can then find a linear relation between the input and output operators, which allows to compute the normalized transmission spectrum (transmitted light with polarization σ', incident light with polarization σ) as

$$T_{\sigma,\sigma'}(\omega) = \frac{\sum_{p'} \langle \hat{\alpha}^{\mathrm{out}\dagger}_{p',\sigma',\mathrm{bot}}(q) \hat{\alpha}^{\mathrm{out}}_{p',\sigma',\mathrm{bot}}(q) \rangle}{\sum_{p} \langle \hat{\alpha}^{\mathrm{in}\dagger}_{p,\sigma,\mathrm{top}}(q) \hat{\alpha}^{\mathrm{in}}_{p,\sigma,\mathrm{top}}(q) \rangle}. \quad \text{(S6)}$$

We assume THz radiation coming from the top reservoir and populating all photonic modes equally, i.e., $\langle \hat{\alpha}^{\mathrm{in}\dagger}_{p,\sigma,\mathrm{top}}(q) \hat{\alpha}^{\mathrm{in}}_{p,\sigma,\mathrm{top}}(q) \rangle$ does not depend on p nor σ, while $\langle \hat{\alpha}^{\mathrm{in}\dagger}_{p,\sigma,\mathrm{bot}}(q) \hat{\alpha}^{\mathrm{in}}_{p,\sigma,\mathrm{bot}}(q) \rangle = 0 \ \forall (p,\sigma)$ and $\langle \hat{\beta}^{\mathrm{in}\dagger}(\boldsymbol{\rho},q) \hat{\beta}^{\mathrm{in}}(\boldsymbol{\rho},q) \rangle = 0 \ \forall \boldsymbol{\rho}$. The output expectation value $\langle \hat{\alpha}^{\mathrm{out}\dagger}_{p,\sigma,\mathrm{bot}}(q) \hat{\alpha}^{\mathrm{out}}_{p,\sigma,\mathrm{bot}}(q) \rangle$ depends on the decay rates of each photonic modes $\Gamma_{p,\sigma} = \omega_p / Q_{p,\sigma} \equiv \pi \kappa^2_{p,\sigma}(q_0) \rho(\omega)$, with $Q_{p,\sigma}$ the quality factor of the cavity mode (p,σ) as computed in FDTD (MEEP implementation [5]) without the 2DEG, and on the CR excitation decay rate $\Gamma_{\mathrm{c}} \equiv \pi \widetilde{\kappa}^2(\boldsymbol{\rho}, \widetilde{q}_0) \rho(\omega)$. The latter corresponds to the intrinsic CR decay rate. The quality factors of the photonic modes were obtained by fitting the peaks in transmittance spectra with Lorentzian functions. However, deviations from pure Lorentzian line shapes were clearly observed (especially for low-Q modes), similar to a previous report for 0D-PCCs [6], which has been attributed to dissipation (radiative leakage of the cavity modes) [7]. Note that the CR decay rate is suppressed when the 2DEG is placed inside a high-Q cavity [8]. As a good estimate, we used the CR decay rate obtained from previous experiments with a 1D-PCC ($\Gamma_{\mathrm{c}}/2\pi = 5.7\,\mathrm{GHz}$) [8]. In the previous equations, q_0 and $\widetilde{q}_0$ are solutions of $\omega - w_{p,\sigma}(q) = 0$ and $\omega - \widetilde{w}(q,\boldsymbol{\rho}) = 0$, respectively, and $\rho(\omega)$ is the effective density of states of the reservoirs, which are assumed to be Markovian, i.e., $\rho(\omega)$ is supposed to be frequency-independent in the range of interest.

In order to further characterize the full and decoupled models discussed in the main text, we introduce a toy model with two cavity modes and one component for the electric field, one polarization, and continuously tunable spatial overlap between the cavity modes. The toy model Hamiltonian reads

$$\begin{aligned}H =& \sum_p \hbar\omega_p \hat{a}_p^\dagger \hat{a}_p + \hbar\omega_c \int d\boldsymbol{\rho}\, \hat{b}^\dagger(\boldsymbol{\rho})\hat{b}(\boldsymbol{\rho}) \\ &+ \sum_p \int \frac{d\boldsymbol{\rho}}{a} \hbar g_p(\boldsymbol{\rho}) \left[\hat{b}(\boldsymbol{\rho}) + \hat{b}^\dagger(\boldsymbol{\rho})\right] \left(\hat{a}_p + \hat{a}_p^\dagger\right) \\ &+ \sum_{p,p'} \int \frac{d\boldsymbol{\rho}}{a^2} \frac{\hbar g_p(\boldsymbol{\rho}) g_{p'}(\boldsymbol{\rho})}{\omega_c} \left(\hat{a}_p + \hat{a}_p^\dagger\right)\left(\hat{a}_{p'}^\dagger + \hat{a}_{p'}\right), \end{aligned} \tag{S7}$$

with the coupling strength $g_p(\boldsymbol{\rho}) = E_p(\boldsymbol{\rho})\sqrt{e^2\omega_c n_e/(4\varepsilon_0 m_{\text{eff}}\omega_p a)}$, and the in-plane mode profiles

$$\begin{aligned}E_1(\boldsymbol{\rho}) &= \sin\left(\frac{2\pi x}{a}\right)\sin\left(\frac{2\pi y}{a}\right) \\ E_2(\boldsymbol{\rho}) &= \sin\left(\frac{2\pi x}{a} + \frac{(1-\epsilon)\pi}{2}\right)\sin\left(\frac{2\pi y}{a} + \frac{(1-\epsilon)\pi}{2}\right). \end{aligned} \tag{S8}$$

The parameter $\epsilon \in [0,1]$ allows us to artificially tune the spatial overlap between the two cavity modes. As ϵ is increased from 0 (no overlap) to 1 (perfect overlap), the splitting between the UP of the first mode $p = 1$ and the LP of the second one $p = 2$ becomes narrower and vanishes at $\epsilon = 1$.

The Hamiltonian (S7) can be written in terms of the CR excitation operators

$$\hat{b}_{\mathbf{G}} = \int \frac{d\boldsymbol{\rho}}{a} \hat{b}(\boldsymbol{\rho}) e^{-i\mathbf{G}\cdot\boldsymbol{\rho}}, \tag{S9}$$

decomposed over the set of reciprocal lattice vectors $G_j = m_j \times 2\pi/a$ ($m_j = 0, 1, 2, ...$ and $j = x, y$) as

$$\begin{aligned}H =& \sum_p \hbar\omega_p \hat{a}_p^\dagger \hat{a}_p + \hbar\omega_c \sum_{\mathbf{G}} \hat{b}_{\mathbf{G}}^\dagger \hat{b}_{\mathbf{G}} \\ &+ \sum_{p,\mathbf{G}} \hbar g_p(\mathbf{G}) \left[\hat{b}_{-\mathbf{G}} + \hat{b}_{\mathbf{G}}^\dagger\right] \left(\hat{a}_p + \hat{a}_p^\dagger\right) \\ &+ \sum_{p,p'} \sum_{\mathbf{G}} \frac{\hbar g_p(\mathbf{G}) g_{p'}(\mathbf{G})}{\omega_c} \left(\hat{a}_p + \hat{a}_p^\dagger\right)\left(\hat{a}_{p'}^\dagger + \hat{a}_{p'}\right), \end{aligned} \tag{S10}$$

with $g_p(\mathbf{G}) = \int \frac{d\boldsymbol{\rho}}{a^2} g_p(\boldsymbol{\rho}) e^{-i\mathbf{G}\cdot\boldsymbol{\rho}}$. The only non-vanishing Fourier coefficients $g_p(\mathbf{G})$ of the mode profile functions (S8) correspond to $m_x = \pm 1$ and $m_y = \pm 1$. The Hamiltonian (S10) can be put in the diagonal form $H = \sum_\lambda \omega_\lambda \hat{p}_\lambda^\dagger \hat{p}_\lambda$, with the polariton

modes

$$\hat{p}_\lambda = \sum_p X_p^\lambda \hat{a}_p + \sum_{\mathbf{G}} W_{\mathbf{G}}^\lambda \hat{b}_{\mathbf{G}} + \sum_p \widetilde{X}_p^\lambda \hat{a}_p^\dagger + \sum_{\mathbf{G}} \widetilde{W}_{\mathbf{G}}^\lambda \hat{b}_{-\mathbf{G}}^\dagger . \tag{S11}$$

Here λ takes 6 values, including a LP (lowest eigenvalue), an UP (largest eigenvalue), and 4 intermediate eigenvalues (MPs) lying in between the LP and the UP (see Supplementary Fig. 1a).

By inverting the transformation (S11), one finds that the correlations between the cavity modes in the state $|\{n_\lambda\}\rangle$ (containing n_λ polaritons in the mode λ) read

$$\langle\{n_\lambda\}|\, \hat{a}_p^\dagger \hat{a}_{p'} \,|\{n_\lambda\}\rangle = \sum_\lambda \left(\widetilde{X}_p^\lambda\right)^* \widetilde{X}_{p'}^\lambda \,(n_\lambda + 1) + \sum_\lambda X_p^\lambda \left(X_{p'}^\lambda\right)^* n_\lambda . \tag{S12}$$

In the polariton vacuum, $n_\lambda = 0\ \forall\lambda$, the only contribution is the first term in the right-hand side of Eq. (S12), which depends on the anomalous coefficients $\widetilde{X}_p^\lambda$. As a hallmark of the USC regime, the latter are expected to be governed by the USC figure of merit (FOM). In particular, we find that the intermode ground-state correlations are well captured by the FOM

$$\eta_{12} = \sqrt{\frac{\int (d\boldsymbol{\rho}/a^2)\, g_1(\boldsymbol{\rho}) g_2(\boldsymbol{\rho})}{\omega_c(\omega_1 + \omega_2)/2}} \tag{S13}$$

introduced in the main text (Supplementary Fig. 1b). On the other hand, the correlations between the cavity modes in the excited states ($n_\lambda \neq 0$) also depend on the coefficients X_p^λ (second term in the right-hand side of Eq. (S12)), which correspond to the weights of the polariton mode λ onto the different cavity modes p. The weights of the MPs onto the modes $p = 1, 2$ are displayed in Supplementary Fig. 1c as a function of the frequency difference $\omega_2 - \omega_1$, showing that the intermode correlations in the excited states are expected to be enhanced in the SSC.

S1.2 Contributions of the different terms in the full Hamiltonian to the ground-state correlations

Supplementary Figure 2 shows the contribution of the ground-state correlations from different terms in the Hamiltonian. The $\hat{H}_{\mathrm{int}}$ term provides the magnetic field dependence of the ground-state correlation (Supplementary Fig. 2a). The $\hat{H}_{A^2}$ term provides the baseline of the ground-state correlation with the full Hamiltonian (Supplementary Fig. 2b), supporting the claim that the $\eta_{pp',j}$ is a suitable FOM for ultrastrong photon–photon coupling. The ground-state correlations are zero if the antiresonant part of the $\hat{H}_{\mathrm{int}}$ and $\hat{H}_{A^2}$ terms are neglected (Supplementary Fig. 2c). The ground-state correlation will be overestimated if the resonant part of the $\hat{H}_{\mathrm{int}}$ and $\hat{H}_{A^2}$ terms are neglected (Supplementary Fig. 2d).

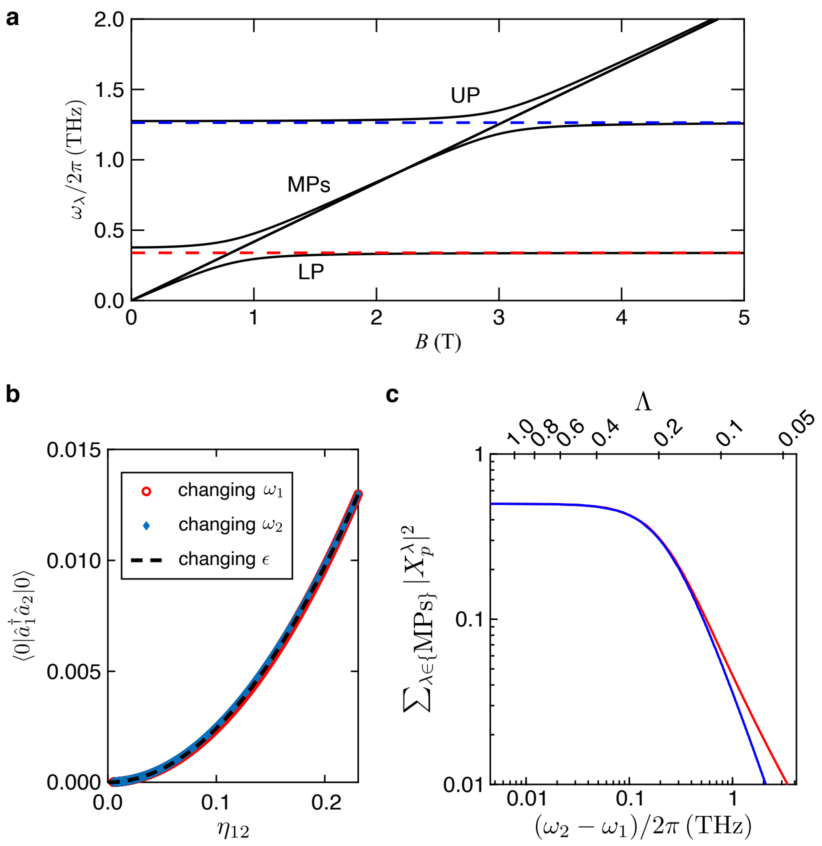

Supplementary Fig. 1 Polariton dispersion, ground-state correlations, and cavity photon weights of a toy model with two cavity modes and continuously tunable spatial overlap. **a**, Polariton dispersion (black lines) as a function of the magnetic field B in the case of perfect overlap of the cavity modes ($\epsilon = 1$), showing an S-shaped MP. The red and blue dashed lines correspond to ω_1 and ω_2, respectively. **b**, Scaling of the intermode correlations with the off-diagonal coupling strength η_{12}. The red circles and blue diamonds are obtained by tuning the frequency $\omega_1 = 2\pi \times [0.339, 1.8]$ THz (while keeping $\omega_2 = 2\pi \times 0.384$ THz and $\epsilon = 1$) and $\omega_2 = 2\pi \times [0.384, 1.8]$ THz (while keeping $\omega_1 = 2\pi \times 0.339$ THz and $\epsilon = 1$), respectively. The black dashed line is obtained by tuning ϵ between 0 and 1, with $\omega_1 = 2\pi \times 0.339$ THz and $\omega_2 = 2\pi \times 0.384$ THz. The magnetic field is set to $B = 0.81$ T, for which $\omega_c = \omega_1$. The overlap of all traces confirms that the intermode correlations at the ground state are governed by the FOM η_{12}. **c**, Weights of the MPs, $\sum_{\lambda\in\{\text{MPs}\}} |X_p^\lambda|^2$, onto the different cavity modes p as a function of the frequency difference $\omega_2 - \omega_1$ with the SSC FOM Λ on top. Here ω_2 is increased while keeping $\omega_1 = 2\pi \times 0.339$ THz and the overlap parameter $\epsilon = 1$ fixed. For each ω_2, the magnetic field is adjusted to the value corresponding to the inflexion point where a MP crosses the CR (see **a**).

S1.3 Vacuum fluctuations and localization of the cavity modes

The vacuum fluctuations of the photonic modes shown in the main text were computed as follows: The expectation value of the electric energy density along z in the vacuum

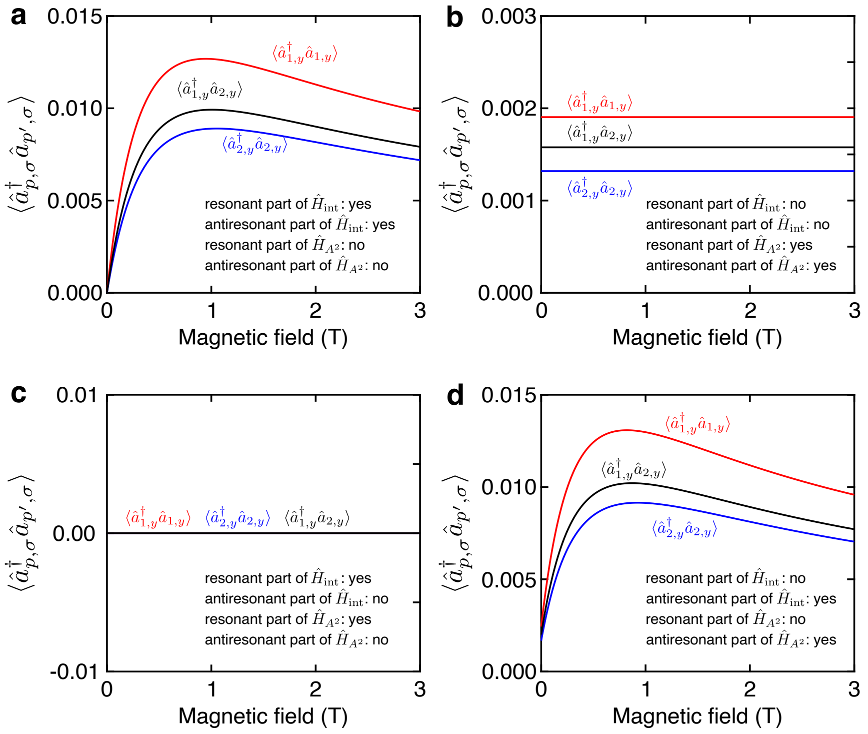

Supplementary Fig. 2 Contribution of the ground-state correlation from different terms in the full Hamiltonian. a–d, Calculated ground-state correlations $\langle \hat{a}^\dagger_{p,j}\hat{a}_{p',j}\rangle$ for TE modes (**a**) with only the $\hat{H}_{\rm int}$ term, (**b**) with only the $\hat{H}_{A^2}$ term, (**c**) with only the resonant part of the $\hat{H}_{\rm int}$ and $\hat{H}_{A^2}$ terms, and (**d**) with only the anti-resonant part of the $\hat{H}_{\rm int}$ and $\hat{H}_{A^2}$ terms.

state $|0\rangle$ is $\langle I(z)\rangle = \int d\boldsymbol{\rho}\, \varepsilon_0\varepsilon(\boldsymbol{\rho},z)\sum_{j=x,y,z}\langle 0|\,\hat{E}_j^2(\boldsymbol{\rho},z)\,|0\rangle/2$, with the component j of the electric field operator

$$\hat{E}_j(\boldsymbol{\rho},z) = i\sum_{p,\sigma}\sqrt{\frac{\hbar\omega_p}{2\varepsilon_0 a^3}}E_{p,\sigma,j}(\boldsymbol{\rho},z)\left(\hat{a}_{p,\sigma}-\hat{a}^\dagger_{p,\sigma}\right). \tag{S14}$$

The contribution of the cavity mode p with polarization σ to that expectation value [i.e., $\langle I(z)\rangle = \sum_{p,\sigma}\langle I_{p,\sigma}(z)\rangle$] thus reads

$$\langle I_{p,\sigma}(z)\rangle = \frac{\hbar\omega_p}{4a}\int\frac{d\boldsymbol{\rho}}{a^2}\,\varepsilon(\boldsymbol{\rho},z)\sum_{j=x,y,z}E^2_{p,\sigma,j}(\boldsymbol{\rho},z). \tag{S15}$$

Note that the integral runs over a woodpile unit cell of surface $a^2 = (333\,\mu\text{m})^2$.

The variance of the in-plane electric field (at the vertical location of the 2DEG) in the vacuum state, $\langle E^2(\boldsymbol{\rho})\rangle = \langle 0|\,\hat{E}_x^2(\boldsymbol{\rho},z_{\rm 2DEG})\,|0\rangle + \langle 0|\,\hat{E}_y^2(\boldsymbol{\rho},z_{\rm 2DEG})\,|0\rangle$, can be

computed in a similar way, i.e., by writing $\langle E^2(\boldsymbol{\rho})\rangle = \sum_{p,\sigma}\langle E^2_{p,\sigma}(\boldsymbol{\rho})\rangle$, with

$$\langle E^2_{p,\sigma}(\boldsymbol{\rho})\rangle = \frac{\hbar\omega_p}{2\varepsilon_0 a^3}\left[E^2_{p,\sigma,x}(\boldsymbol{\rho}, z_{\mathrm{2DEG}}) + E^2_{p,\sigma,y}(\boldsymbol{\rho}, z_{\mathrm{2DEG}})\right]. \tag{S16}$$

The quantity plotted on Fig. 2c–f in the main text is the standard deviation $\sqrt{\langle E^2_{p,\sigma}(\boldsymbol{\rho})\rangle}$.

The $j = x$ and $j = y$ components of the variance of the in-plane electric field in the vacuum state are displayed in Supplementary Fig. 3. It is worthy to note that $\sqrt{\langle E^2_{p,\sigma}(\boldsymbol{\rho})\rangle}$ is mainly polarized along the σ direction. The mode profiles for $\sigma \neq j$ (Supplementary Fig. 3b,d) are much weaker than those for $\sigma = j$ (Supplementary Fig. 3a,c).

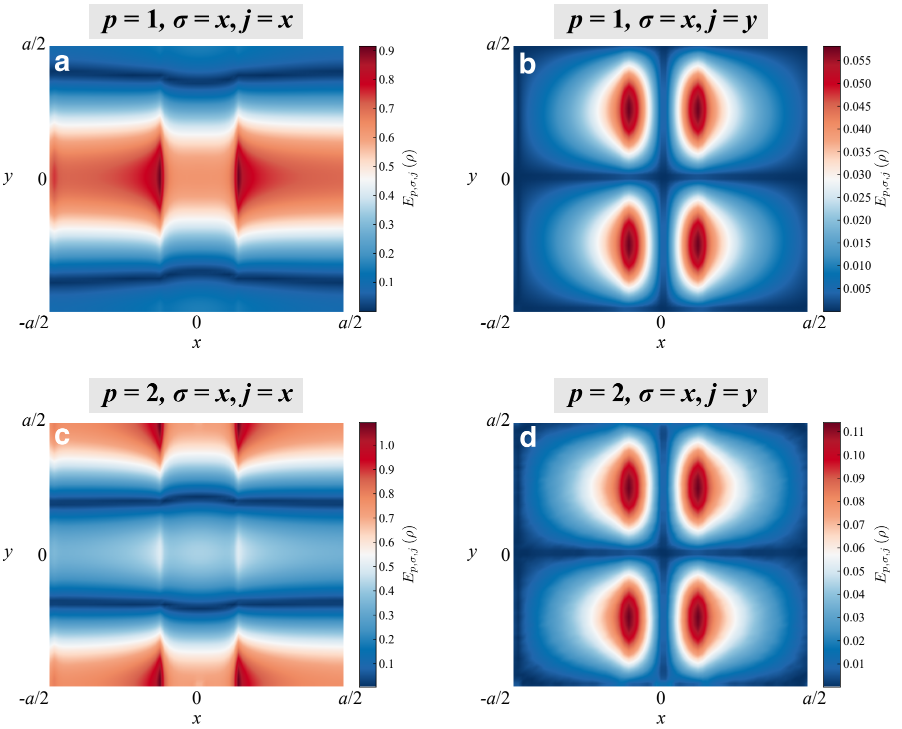

Supplementary Fig. 3 Real-space distribution of electric field in different components. **a**–**d**, The $j = x$ and $j = y$ components of the electric field profile of cavity mode $p = 1$ (**a**–**b**) and mode $p = 2$ (**c**–**d**) for $\sigma = x$.

In order to assess the degree of localization of the different cavity modes in the z direction, one can treat the electric energy density of the mode p with polarization σ

as a probability distribution since

$$\int \frac{d\boldsymbol{\rho}}{a^2} \int \frac{dz}{a}\, \varepsilon(\boldsymbol{\rho}, z) \sum_{j=x,y,z} E^2_{p,\sigma,j}(\boldsymbol{\rho}, z) = 1, \tag{S17}$$

and compute the standard deviation of the electric energy density along z as

$$\sigma_{p,\sigma} = \sqrt{\int \frac{d\boldsymbol{\rho}}{a^2} \int \frac{dz}{a}\, (z - \bar{z}_{p,\sigma})^2\, \varepsilon(\boldsymbol{\rho}, z) \sum_{j=x,y,z} E^2_{p,\sigma,j}(\boldsymbol{\rho}, z)}, \tag{S18}$$

with the mean value

$$\bar{z}_{p,\sigma} = \sqrt{\int \frac{d\boldsymbol{\rho}}{a^2} \int \frac{dz}{a}\, z\, \varepsilon(\boldsymbol{\rho}, z) \sum_{j=x,y,z} E^2_{p,\sigma,j}(\boldsymbol{\rho}, z)}. \tag{S19}$$

Since the photonic modes are localized in the vicinity of the defect layer along the z direction and exhibit discrete translational symmetry in the plane, we compute the spatial profile $E_{p,\sigma,j}(\boldsymbol{\rho}, z)$ in a woodpile unit cell. The integral along z runs over the whole computational cell, which includes air layers on each side of the woodpile structure along the z direction. The standard deviations of the 3D-PCC are $\sigma_{1,x} \approx \sigma_{2,x} \approx \sigma_{1,y} \approx \sigma_{2,y} \approx 0.5a$ (about two Si rods thick), and $\sigma_{3,x} \approx \sigma_{4,x} \approx \sigma_{3,y} \approx \sigma_{4,y} \approx 0.18a$ (less than one Si rod thick). For all cavity modes, more than 95% of the electric energy is located in the 3D-PCC. As expected, the localization of the cavity modes along the z direction increases as the latter are located deeper into the photonic band gap.

S2 Numerical simulations

S2.1 Details of simulations

The mode profiles of the cavity modes induced by a 60 µm-thick bare GaAs layer were calculated by the MEEP software [5]. The electric field was assumed to be constant over the thickness $d \simeq 2$ µm of the QW heterostructure. The transmittance spectrum of the bare cavity in the main text was simulated by using the COMSOL Multiphysics software. We used the permittivity $\varepsilon = 11.6964$ for the silicon layers, and $\varepsilon = 12.96$ for the GaAs layer. For the simulations with the 2DEG layer in the main text, because the woodpile structure exhibits mirror symmetries, only 1/4 of the unit cell of the woodpile structure was considered in the geometry to reduce the simulation time. A transition boundary condition with an effective thickness, $d = 2$ µm, was used to emulate the MQW structure. A gyrotropic permittivity tensor was used to describe the complex permittivity of the 2DEG layer at different magnetic fields:

$$\tilde{\varepsilon} = \begin{pmatrix} \varepsilon_{xx}(\omega) & \varepsilon_{xy}(\omega) & 0 \\ -\varepsilon_{xy}(\omega) & \varepsilon_{xx}(\omega) & 0 \\ 0 & 0 & \varepsilon_{zz} \end{pmatrix}, \tag{S20}$$

with

$$\varepsilon_{xx}(\omega) = \varepsilon_{\mathrm{bg}} - \frac{\omega_{\mathrm{pl}}^2(\omega - i\gamma)}{\omega d[(\omega - i\gamma)^2 - \omega_{\mathrm{c}}^2]}, \tag{S21}$$

$$\varepsilon_{xy}(\omega) = \frac{-i\omega_{\mathrm{pl}}^2\omega_{\mathrm{c}}}{\omega d[(\omega - i\gamma)^2 - \omega_{\mathrm{c}}^2]}, \tag{S22}$$

$$\varepsilon_{zz} = \varepsilon_{\mathrm{bg}}, \tag{S23}$$

where $\varepsilon_{\mathrm{bg}} = 12.96$ is the background permittivity, $\omega_{\mathrm{pl}} = \sqrt{n_e e^2/(\varepsilon_0 m_{\mathrm{eff}})}$ is the plasma frequency and $\omega_{\mathrm{c}} = eB/m_{\mathrm{eff}}$ is the cyclotron frequency.

S2.2 Optimized thickness of the defect layer in numerical calculations

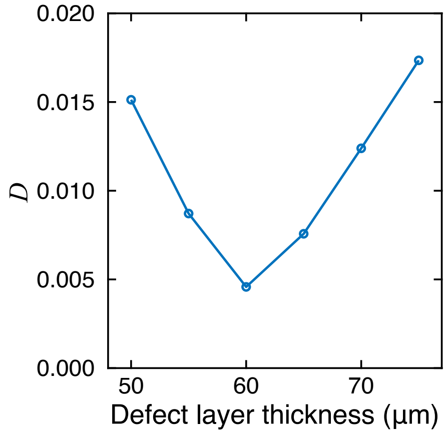

Supplementary Fig. 4 Optimization of the thickness of the defect layer. Deviation, D, as a function of the defect layer thickness. D reaches its minimum when the defect layer is 60-μm-thick.

Due to imperfections in mechanical polishing, the thickness of the GaAs substrate of the 2DEG layer in the experiment was not perfectly homogeneous. To determine the suitable thickness of the substrate for numerical simulations, we ran simulations in the presence of the 2DEG layer at $B = 0$, 1.5, 2.5, and 7 T with different thicknesses of the GaAs substrate. We calculated the deviations of the polariton frequencies for $\sigma = x$ and $\sigma = y$ modes between the experiment and simulation, $\Delta f_{P,B,\sigma}$, where $P = \{\mathrm{UP}, \mathrm{LP1}, \mathrm{LP2}\}$, at each B. Finally, we calculated the root mean square of the total deviation,

$$D = \sqrt{\frac{\sum_{P,B,\sigma} \Delta f_{P,B,\sigma}^2}{2N}}, \tag{S24}$$

where $N = 13$ is the number of data points. The optimized thickness of the defect layer that gives the minimum deviation is 60 µm (including the 2-µm-thick MQW layer), as shown in Supplementary Fig. 4.

S2.3 Simulations with different ranges of time delays

We investigated the transmittance spectra of the system with different ranges of time delays in FDTD simulations (Lumerical). As shown in Supplementary Fig. 5, in order to resolve modes 3 and 4 with high Q-factors in the spectrum, a long time-domain trace is needed. The amplitudes of modes 3 and 4 are weak for a time-domain trace up to 200 ps.

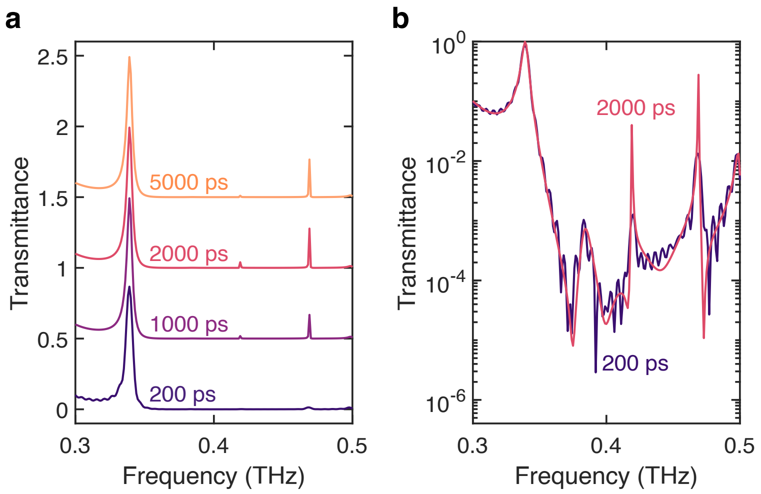

Supplementary Fig. 5 Simulations with different lengths of time window. a, Transmittance spectra of a bare 3D-PCC with a series of time windows obtained from simulations. The peaks corresponding to the third and fourth cavity modes become pronounced when the time window is longer than 1000 ps. **b**, Transmittance spectra in (**a**) that are plotted in logarithmic scale.

S2.4 Simulations of a bare 3D-PCC with varying defect layer thicknesses

We simulated the transmittance spectrum of a bare 3D-PCC as a function of defect layer thickness, as shown in Supplementary Fig. 6. The results demonstrate that the overall spectral features of the cavity, including modes 1–4, remain robust despite variations in the defect layer thickness. The mode frequencies exhibit slight shifts with changes in the defect layer thickness.

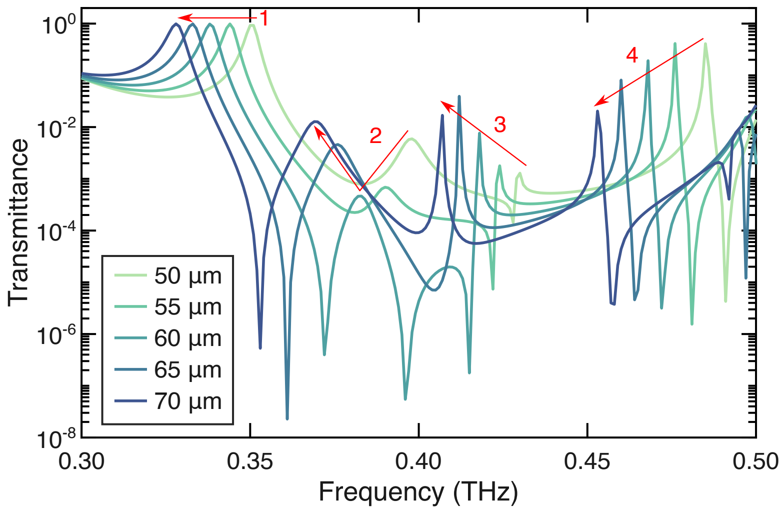

Supplementary Fig. 6 Simulated transmittance spectra of a bare 3D-PCC as a function of defect layer thickness. The transmittance spectra of a bare 3D-PCC are simulated for defect layer thicknesses ranging from 50 µm to 70 µm. Red arrows mark the shifts of modes 1–4.

S3 Experiment

S3.1 Noise floor of the THz setup

The noise measurement was performed when the THz beam path was blocked [9]. We repeated the noise measurement three times and took the root-mean-square of the THz time-domain trace. The THz signal and the noise level are shown in Supplementary Fig. 7a. Both traces are normalized to the maximum value of the THz signal. The noise curve was smoothed to avoid spikes. In the frequency range of interest (0.3–0.45 THz), the noise is lower than 10^{-5} (Supplementary Fig. 7b). The dynamic range of the setup, which is defined as the ratio between the signal and noise level, is shown in Supplementary Fig. 7c. The dynamic range is always higher than 50 dB in this frequency range.

S3.2 Bare cyclotron resonance

We performed THz transmission measurements on the MQW sample without the cavity at various B. Each transmittance spectrum is normalized to the spectrum taken at $B = 7\,\mathrm{T}$, where the CR shifts beyond our bandwidth to high frequencies. Supplementary Figure 8a displays representative data, showing a transmittance dip corresponding to the CR blueshift with increasing B. The dip was analyzed by fitting the data with a Lorentzian function. A linear fit to the peak frequencies extracted from the Lorentzian fits indicates an electron effective mass, m_{eff}, of approximately $0.07\,m_e$ (see Supplementary Fig. 8b), which is consistent with previous reports [8].

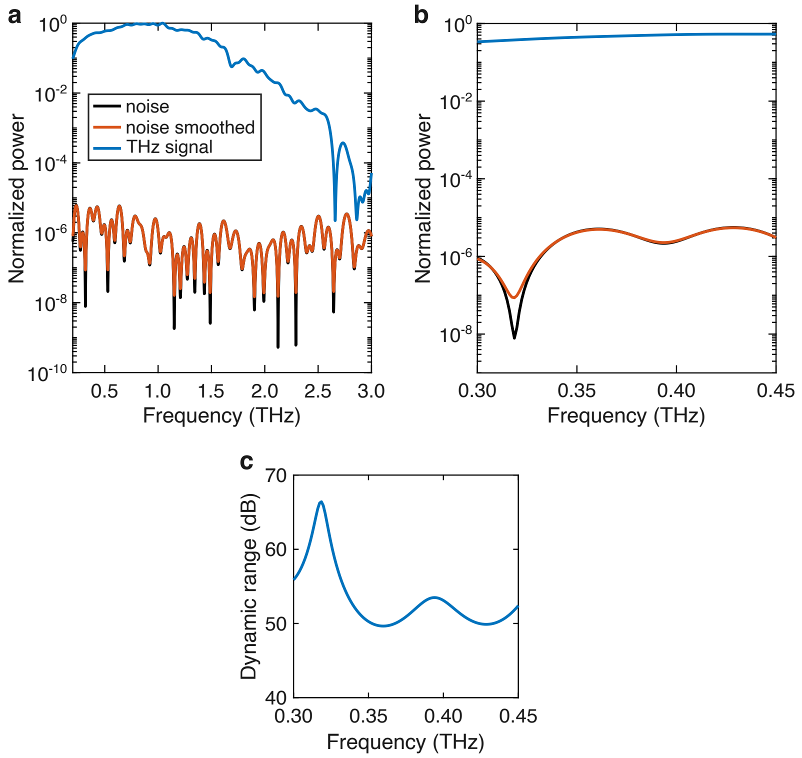

Supplementary Fig. 7 Noise floor analysis of the THz setup. **a**, Power spectra of the THz signal and the root-mean-square noise signal, both normalized to the maximum value of the THz signal. The "noise smoothed" denote the noise spectrum smoothed using a Gaussian filter. **b**, A magnified view of panel (**a**). **c**, Dynamic range of the THz setup.

S3.3 Microscope images

Microscope images of individual patterned silicon wafers and a stacked structure are shown in Supplementary Fig. 9.

S3.4 Transmittance spectra of the bare 3D-PCC

We performed transmission measurements on the 3D photonic crystals with and without a defect layer (a bare GaAs substrate without the 2DEG layer) to examine the quality of the fabricated cavity. As shown in Supplementary Fig. 10a, the transmittance spectra of the 3D photonic crystal without a defect layer exhibit excellent agreement with simulations for frequencies below 0.45 THz. These spectra were obtained using time-domain traces truncated at 70 ps and 300 ps for data analysis. As discussed in the main text, while the experimental data with a 300 ps time window

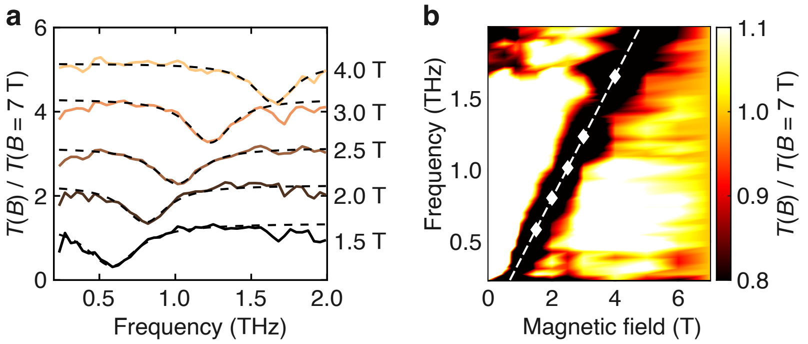

Supplementary Fig. 8 Bare CR. **a**, Normalized transmittance spectra (T) of the MQW sample measured at various magnetic fields (B), referenced to the transmittance at $B = 7\,\mathrm{T}$. The traces are vertically offset for clarity. The dashed lines represent Lorentzian fits to the data. **b**, Color plot of $T(B)$, where the dark region corresponds to the CR. White diamonds represent the peak frequencies extracted from panel (**a**), with the dashed line indicating a linear fit to these frequencies.

provides higher frequency resolution, it also exhibits artificial rapid oscillations in the frequency spectra. These oscillations are caused by unwanted back-reflections of the THz pulses collected at longer delays (the Fabry–Pérot effect), see further details in sec. S3.5. The observed photonic band gap matches well with the simulation result, except for the upper band edge, which appears at a slightly higher frequency in the experimental data. This discrepancy may arise from variations in layer thickness or minor deviations in rod widths during the fabrication process. Importantly, the multi-mode light–matter coupling discussed in the main text occurs below 0.45 THz, where experimental data and simulations exhibit good agreement. Supplementary Figure 10b demonstrates that the transmittance spectra of the 3D photonic crystal without a defect layer are degenerate for orthogonal polarizations, further confirming the high quality of the fabricated structure.

For the measurements on a bare 3D-PCC with a defect layer, we polished a bare GaAs wafer (without the 2DEG layer) down to ∼85 µm. The thickness of the GaAs wafer differs from the thickness of the actual GaAs QW sample due to the inaccuracy of the polishing process. Although this bare GaAs substrate is thicker, the thickness discrepancy only caused shifts of photonic mode frequencies, as shown in Supplementary Fig. 6. The experimental spectra of the bare 3D-PCC align well with the simulation, see Supplementary Fig. 11. When a defect layer is introduced, the overall transmission amplitude decreases due to surface roughness and imperfections introduced to the defect layer during mechanical polishing. For this reason, the simulation was scaled by a factor of 0.6 to match the experimental data.

Importantly, these imperfections primarily reduce the transmission amplitude without significantly altering the spectral features. For instance, as shown in Supplementary Fig. 11, two peaks near the frequencies of modes 1 and 2 in the simulation are observed in the experimental data using a time-domain trace truncated at 70 ps (red trace). Modes 3 and 4 are not resolved in experiments due to the limited frequency resolution. It should be noted that the actual linewidth of the peaks is narrower,

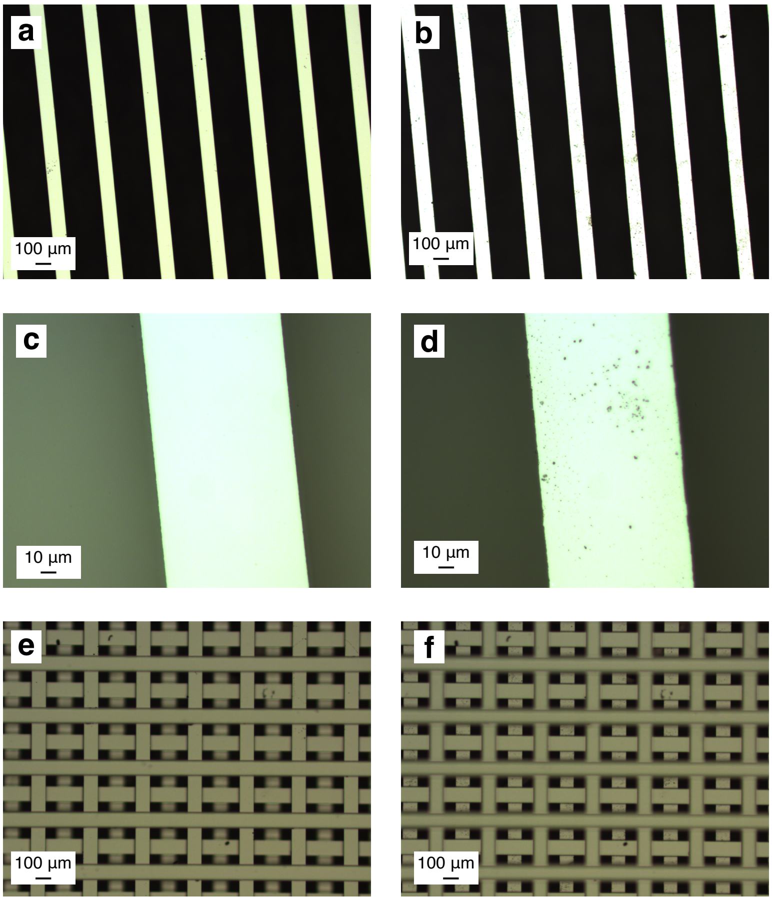

Supplementary Fig. 9 Optical microscope images of 3D photonic crystal structures. a–b, Low-magnification images of two different patterned silicon wafers. **c–d**, High-magnification views of the wafers in panel (**a**) and (**b**). **e–f**, Microscope images of a unit cell of the woodpile structure consisting of four stacked layers, captured at different focal planes.

but the intrinsic frequency resolution of the 70 ps data is limited ($\Delta f \sim 0.014$ THz). Extending the time window to 300 ps increases the maximum amplitude of the peaks and decreases their linewidths (blue trace) due to higher intrinsic frequency resolution. However, the spectra obtained with the 300 ps time window also exhibit artificial oscillations caused by back-reflections of the THz pulses. The spectral shape of the peak corresponding to mode 1 aligns with the simulation result when the oscillations are disregarded. Furthermore, the peak corresponding to mode 2 appears to split due to the influence of these rapid oscillations. Despite these artifacts, the overall spectra features remain consistent with the simulation results, apart from the reduced transmission amplitude. In addition, the measured transmittance spectra for polarizations

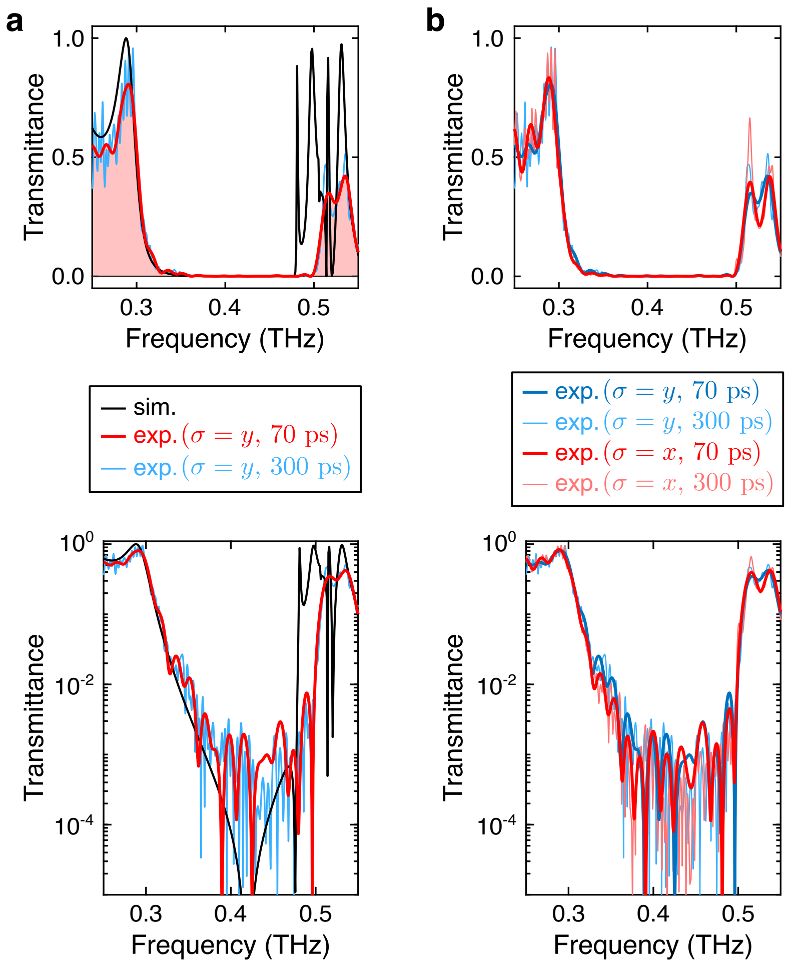

Supplementary Fig. 10 THz transmission spectra of a fabricated 3D photonic crystal without a defect layer. a, Transmittance spectra of a woodpile structure with 2 unit cells, shown on both (top) a linear scale and (bottom) a logarithmic scale. The black curve represents the simulation results, while the red and blue curves denote experimental data for polarizations $\sigma = y$ using time windows of 70 ps and 300 ps, respectively. **b**, Transmittance spectra of the 3D photonic crystal for orthogonal polarizations. The bottom panel shows the corresponding logarithmic-scale plot.

$\sigma = x, y$, as shown in Supplementary Fig. 12, confirm that the cavity mode frequencies of the bare 3D-PCC are degenerate in both polarizations.

S3.5 Extraction of peak frequencies

The linewidth of the polariton branches in the color plot presented in the main text is limited by the intrinsic frequency resolution of the measurements, as a short time-domain range (33 ps) was used. The intrinsic frequency resolution of the time-domain

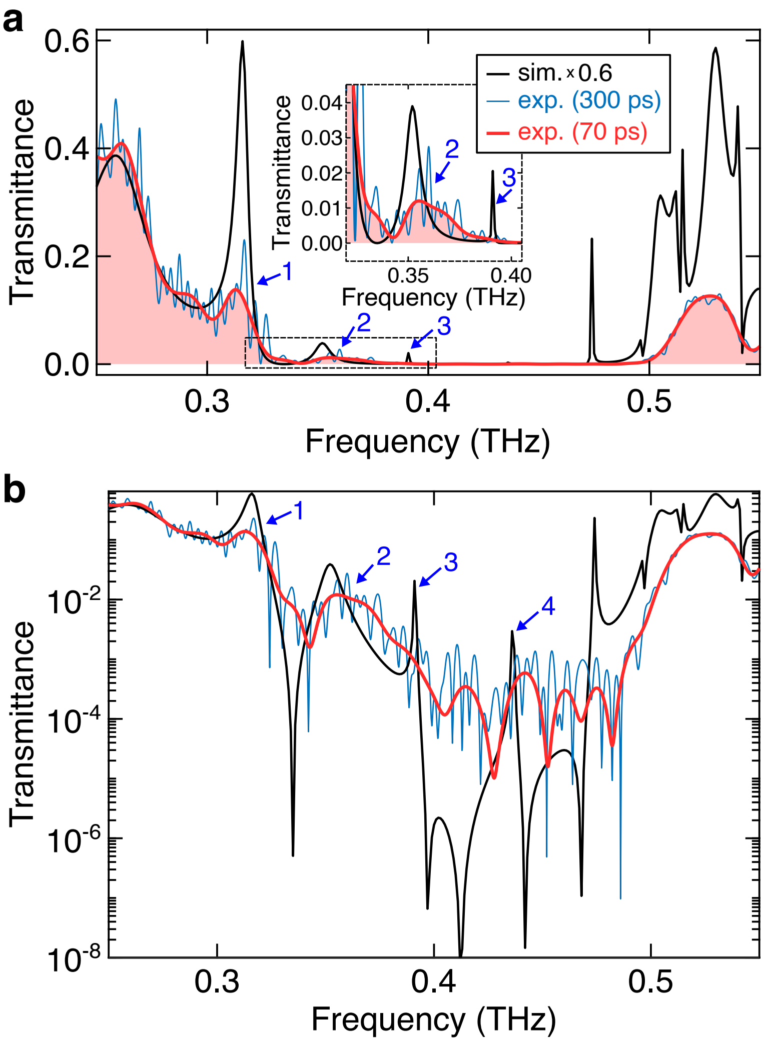

Supplementary Fig. 11 THz transmission spectra of a bare 3D-PCC with an 85-μm-thick defect layer. a–b, Transmittance spectra shown (**a**) on a linear scale and (**b**) on a logarithmic scale. The black curve denotes the simulation results scaled by a factor of 0.6 for clarity, while the red and blue curves depict experimental data processed using Fourier transformation with time windows up to 70 ps and 300 ps, respectively. The rapid oscillations in the 300 ps data are artifacts resulting from back-reflections of the THz pulses. An inset in (**a**) highlights the detailed structure of the low-transmittance region. Modes 3 and 4 are not resolved in the experimental data due to the limited frequency resolution.

data is determined by $1/T$, where T is the length of the time window. To resolve the actual linewidth of the coupled modes in the high-Q 3D-PCC, it is necessary to scan a longer range of time delays during THz-TDS measurements.

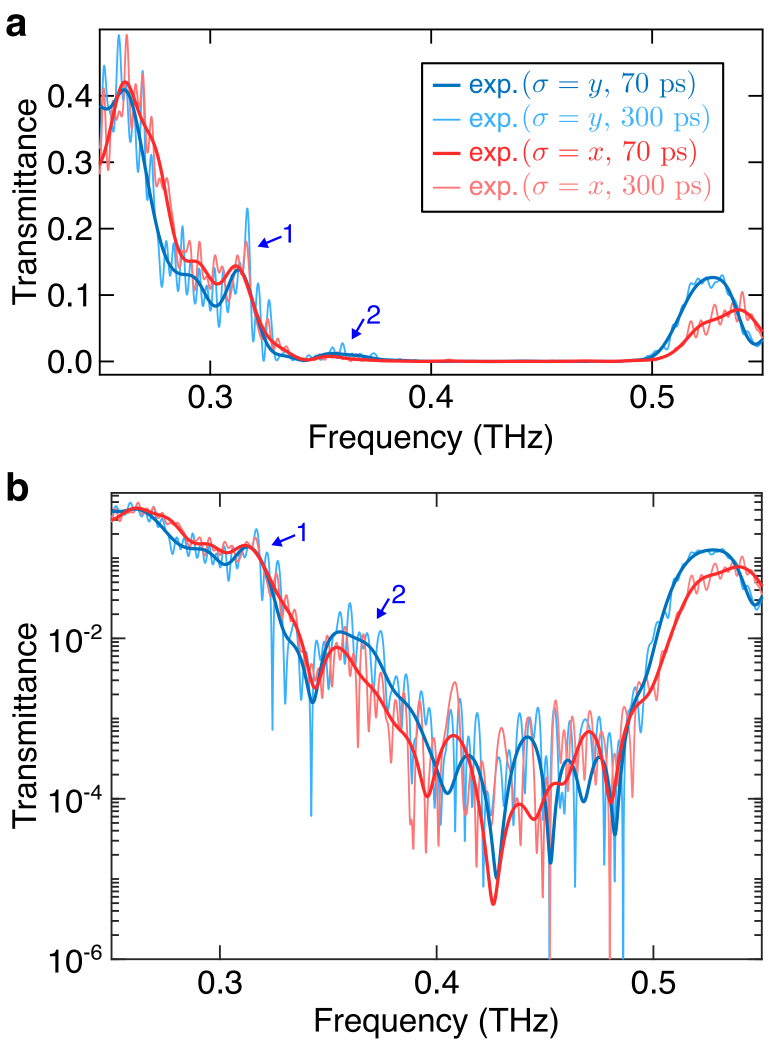

Supplementary Fig. 12 Degenerate cavity modes of a bare 3D-PCC with an 85-µm-thick defect layer for polarizations $\sigma = x, y$. a–b, Transmittance spectra plotted (**a**) on a linear scale and (**b**) on a logarithmic scale. The red and blue curves represent experimental data processed using Fourier transformation with time windows up to 70 ps and 300 ps for $\sigma = x$ and $\sigma = y$, respectively. The cavity modes 1 and 2 are highlighted with blue arrows.

For instance, Supplementary Fig. 13a,b compares experimental transmission spectra obtained with two different ranges of time delays (33 ps and 200 ps, respectively) for $\sigma = x$ polarization at various B. The peaks in Supplementary Fig. 13b, derived from the longer time-domain range, are narrower than those in Supplementary Fig. 13a. However, data collected at long delays unavoidably include back-reflections of THz pulses from several optical components of the system, e.g., cryostat windows and

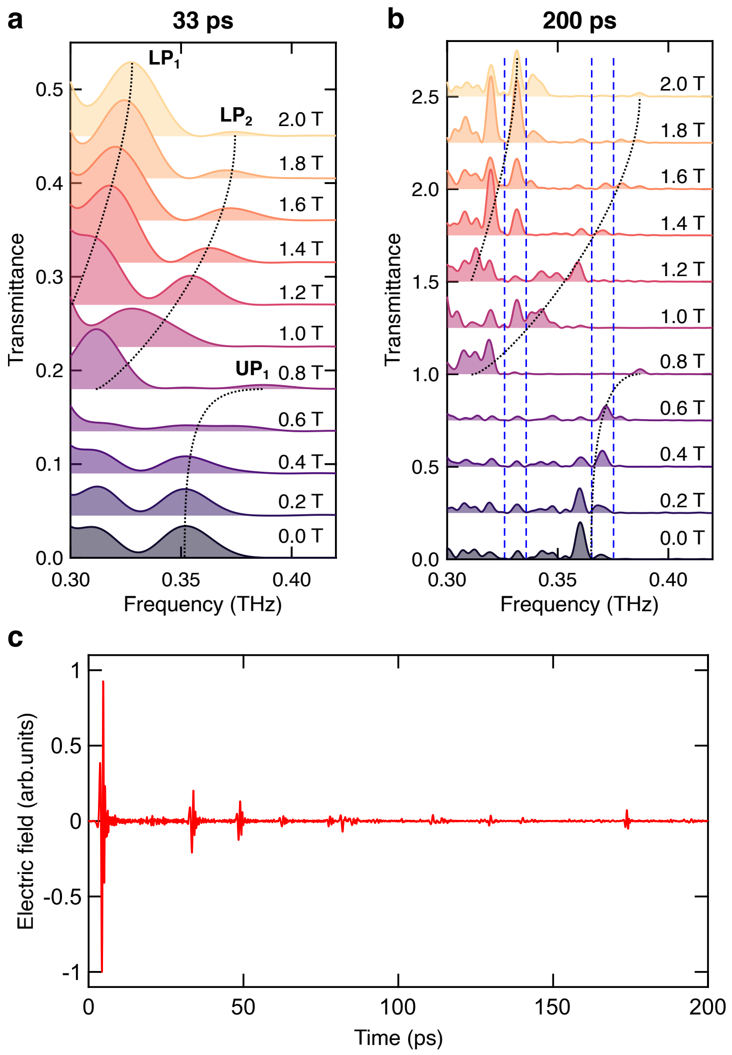

Supplementary Fig. 13 Experimental data with different lengths of the time window. a–b, Transmittance spectra with time windows of up to 33 ps (**a**) and 200 ps (**b**) for $\sigma = x$ polarization at various B. Vertical offsets are applied for clarity. Black dotted curves represent the B-dependent polariton frequency shifts, while blue vertical dashed lines in (**b**) mark some dips induced by Fabry-Pérot modulation, which are unaffected by B. **c**, Temporal waveform of THz radiation measured through an empty aperture without the sample. The observed echoes result from reflections in various optical components, including cryostat windows and crystals.

crystals. Consequently, the time-domain data contains nonuniformly spaced echoes, even in the absence of the sample, as shown in Supplementary Fig. 13c. These echoes lead to multiple artificial dips in the sample's transmittance spectra (the Fabry–Pérot effect), which are unequally spaced in the frequency domain. These artificial dips can

cause spurious peak splitting when spectral peaks overlap with the dips [3]. Importantly, these dips induced by the Fabry–Pérot effect can be identified because their frequencies remain unaffected by B, as indicated by the blue vertical dotted lines in Supplementary Fig. 13b.

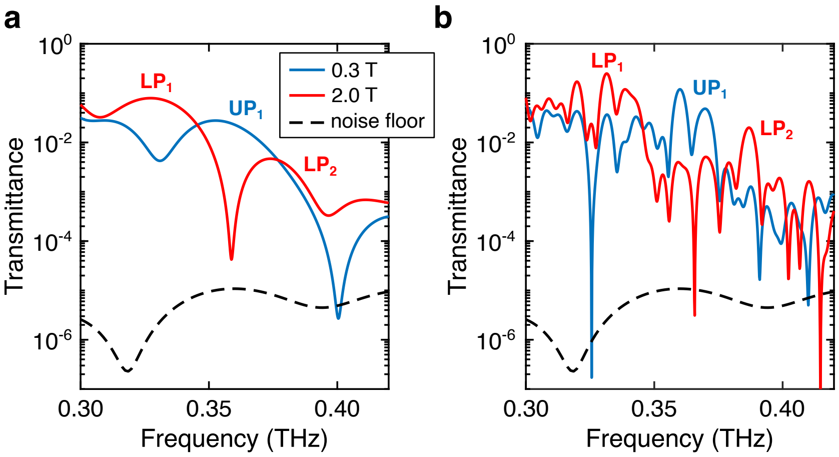

Supplementary Fig. 14 Comparison of experimental transmittance spectra and the noise floor. a–b, Transmittance spectra of the sample at $B = 0.3\,\mathrm{T}$ and $B = 2.0\,\mathrm{T}$ for $\sigma = x$ polarization, using time windows of (**a**) 33 ps and (**b**) 200 ps. The black dashed line represents the noise floor, normalized to the THz signal without a sample, obtained from Fig. 7. The noise floor remains approximately at or below 10^{-5} in this frequency range. Peaks corresponding to polaritons are labeled.

It is important to note that the peaks in the experimental spectra are well above the noise floor level of the setup. As a representative example, Supplementary Fig. 14 compares the experimental transmittance spectra at $B = 0.3\,\mathrm{T}$ and $2.0\,\mathrm{T}$ for $\sigma = x$ polarization. The observed peaks have amplitudes exceeding 10^{-4}, while the noise floor are approximately at or below 10^{-5} within the frequency range of interest.

To determine the peak position from the spectra with Fabry-Pérot modulation, the peaks were fitted using a combination of a Lorentzian peak for the main peak and Gaussian functions for the dips. For example, Supplementary Fig. 15a,b shows the fittings to the UP_1 for $\sigma = x$ polarization at $B = 0.3\,\mathrm{T}$ and $B = 2.0\,\mathrm{T}$, respectively. As a representative example, the extracted Lorentzian peaks for the UP_1 for $\sigma = x$ polarization at various B are shown in Supplementary Fig. 15c. The Lorentzian peak frequencies from these fittings are represented as white dots in the main text. The white dots, extracted from long time-domain traces, show agreement with the color plot extracted from short time-domain traces, demonstrating consistency between the data using different time windows.

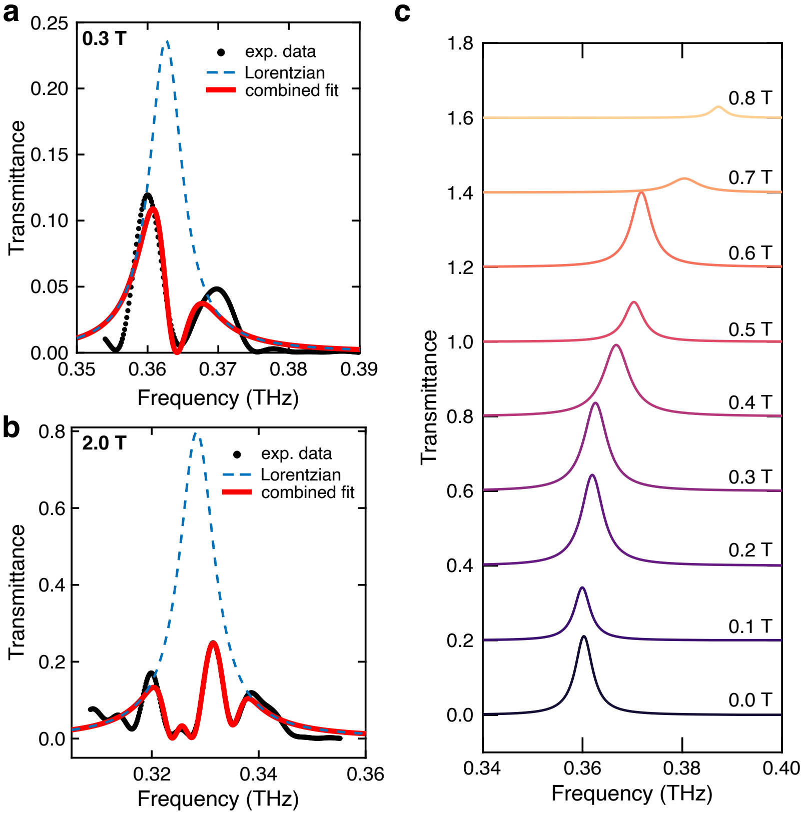

Supplementary Fig. 15 Extraction of peak frequencies from transmittance spectra with a long time-domain waveform. a–b, Fittings to experimental transmittance spectra with a combination of a Lorentzian peak and Gaussian dips for $B = 0.3\,\mathrm{T}$ (**a**) and $B = 2.0\,\mathrm{T}$ (**b**) for $\sigma = x$ modes. **c**, The Lorentzian peaks extracted from the fittings to UP_1 as a function of magnetic field.

S3.6 Error bars of the extracted peak frequencies

We identify two primary sources of uncertainty in the extracted peak frequencies. First, the fitting uncertainty at each B, denoted as Δf_{fit}, is estimated using the 95% confidence interval of the fits. Second, the peak frequency may exhibit slight variations due to system instabilities. These instabilities arise from factors such as laser power fluctuations and minor sample misalignments during measurements. While these variations predominantly affect the transmittance amplitude, the spectral features remain robust, allowing reliable peak frequency extraction. However, slight fluctuations in the extracted peak frequency can still occur.

To evaluate these effects, we plotted the transmittance spectra at $0\,\mathrm{T}$ measured on four different days for $\sigma = x$ polarization (Supplementary Fig. 16a) and compared

the extracted UP_1 frequencies (Supplementary Fig. 16b). The standard deviation of these peak frequencies is treated as the systematic error, Δf_{system}. We assume that Δf_{system} remains constant across all values of B. Finally, the total uncertainty in the peak frequency is expressed as $\Delta f_{\text{peak}} = \sqrt{(\Delta f_{\text{fit}})^2 + (\Delta f_{\text{system}})^2}$.

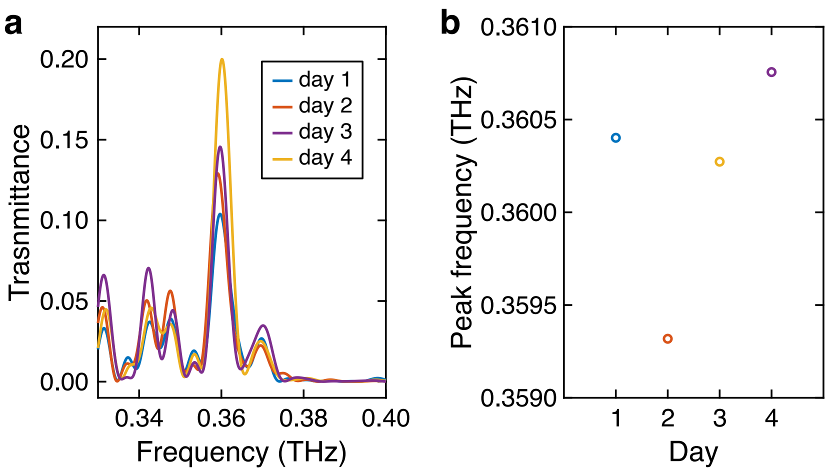

Supplementary Fig. 16 Peak frequency variations caused by system fluctuations. **a**, Transmittance spectra at $B = 0\,\text{T}$ for $\sigma = x$ polarization, measured on different days. A time window of 200 ps was used for the Fourier transformation. **b**, Peak frequencies extracted from the spectra in panel (**a**).

Supplementary References

[1] Hagenmüller, D., De Liberato, S. & Ciuti, C. Ultrastrong coupling between a cavity resonator and the cyclotron transition of a two-dimensional electron gas in the case of an integer filling factor. *Phys. Rev. B* **81**, 235303 (2010).

[2] Hagenmüller, D. All-optical dynamical Casimir effect in a three-dimensional terahertz photonic band gap. *Phys. Rev. B* **93**, 235309 (2016).

[3] Li, X. *et al.* Vacuum Bloch–Siegert shift in Landau polaritons with ultra-high cooperativity. *Nat. Photon.* **12**, 324–329 (2018).

[4] Ciuti, C. & Carusotto, I. Input-output theory of cavities in the ultrastrong coupling regime: The case of time-independent cavity parameters. *Phys. Rev. A* **74**, 033811 (2006).

[5] Oskooi, A. F. *et al.* Meep: A flexible free-software package for electromagnetic simulations by the FDTD method. *Comput. Phys. Commun.* **181**, 687–702 (2010).

[6] Pellegrino, D. *et al.* Non-Lorentzian local density of states in coupled photonic crystal cavities probed by near- and far-field emission. *Phys. Rev. Lett.* **124**, 123902 (2020).

[7] Sauvan, C., Hugonin, J. P., Maksymov, I. S. & Lalanne, P. Theory of the spontaneous optical emission of nanosize photonic and plasmon resonators. *Phys. Rev. Lett.* **110**, 237401 (2013).

[8] Zhang, Q. *et al.* Collective non-perturbative coupling of 2D electrons with high-quality-factor terahertz cavity photons. *Nat. Phys.* **12**, 1005–1011 (2016).

[9] Neu, J. & Schmuttenmaer, C. A. Tutorial: An introduction to terahertz time domain spectroscopy (THz-TDS). *J. Appl. Phys.* **124**, 231101 (2018).
